# Temporal trends in areas at risk for concomitant tuberculosis in a hyperendemic municipality in the Amazon region of Brazil

**DOI:** 10.1186/s40249-020-00732-0

**Published:** 2020-08-10

**Authors:** Alexandre Tadashi Inomata Bruce, Thais Zamboni Berra, Felipe Lima dos Santos, Yan Mathias Alves, Ludmilla Leidianne Limirio Souza, Antônio Carlos Vieira Ramos, Luiz Henrique Arroyo, Juliane de Almeida Crispim, Ione Carvalho Pinto, Pedro Fredemir Palha, Aline Aparecida Monroe, Mellina Yamamura, Regina Célia Fiorati, Ana Carolina Scarpel Moncaio, Dulce Maria de Oliveira Gomes, Ricardo Alexandre Arcêncio

**Affiliations:** 1 Department of Maternal-Infant and Public Health Nursing, University of São Paulo at Ribeirão Preto College of Nursing, Ribeirão Preto, São Paulo, Brazil; 2 Department of Nursing, Federal University of São Carlos, São Carlos, São Paulo, Brazil; 3 Department of Neurosciences and Behavioral Sciences, Ribeirão Preto Medical School of the University of São Paulo, Ribeirão Preto, São Paulo, Brazil; 4 Department of Nursing, Federal University of Catalão, Catalão, Goiás, Brazil; 5 Mathematics Department, University of Évora, Évora, Portugal

**Keywords:** Tuberculosis, Public health, Epidemiology, Spatial analysis, Spatio-temporal analysis

## Abstract

**Background:**

Although preventable and curable, tuberculosis (TB) still occurs in poor or developing countries, mainly in metropolitan regions of larger cities. The disease is a serious public health problem, and is directly linked to social issues. We analyzed temporal trend variations in areas at risk for concomitant TB, and characterized the clinical and epidemiological profiles of cases in a hyperendemic municipality in the Amazon region of Brazil.

**Methods:**

This ecological study was performed in the municipality of Manaus, in northern Brazil. The population comprised cases with concomitant pulmonary and extrapulmonary TB, registered on the Notifiable Diseases Information System (SINAN), between January 1, 2009 and December 31, 2018. For risk cluster detection, spatial and spatiotemporal scanning statistical techniques were used. The Spatial Variation in Temporal Trends (SVTT) approach was used to detect and infer clusters for significantly different time trends.

**Results:**

Between 2009 and 2018, 873 concomitant TB cases were registered in Manaus. By using purely spatial scanning statistics, we identified two risk clusters. The relative risk (RR) of the clusters was 2.21 (95% confidence interval [*CI*]: 1.57–2.88; *P* = 0.0031) and 2.03 (95% *CI*: 1.58–2.58; *P* = 0.0029). Using space-time scanning, we identified a risk cluster with an RR of 3.57 (95% *CI*: 2.84–4.41; *P* = 0.014), between 2017 and 2018. For SVTT analyses, three clusters with spatial variations were detected in the significant temporal trends: SVTT 1 (*P* = 0.042), SVTT 2 (*P* = 0.046) and SVTT 3 (*P* = 0.036).

**Conclusions:**

In Brazil, several TB-determining factors such as race/color, gender, low educational level and low income overlap in needy urban areas and communities, demonstrating that it is unlikely to reach the goals, agreed and launched with the END TB Strategy within the deadlines of international agreements, if there is no reduction in existing inequities determinants and risk of illness in the country.

## Background

Globally, tuberculosis (TB) is the biggest killing infectious disease, outdoing human immunodeficiency virus (HIV) [1]. Caused by *Mycobacterium tuberculosis*, the disease has a long latency period between initial infection and clinical presentation [2]. Although preventable and curable, many TB cases are registered in poor or developing countries, mainly in metropolitan regions of large cities as such, the disease is considered a serious public health problem, directly linked to social issues [1].

Approximately 85% of registered TB cases are pulmonary, due to a predilection of the *Bacillus* for the lungs, however other parts of the body are also affected [3]. Thus, disease signs and symptoms vary depending on the systems or organs affected, therefore the disease is classified according to location, e.g. bones, kidneys, central nervous system, etc. Extrapulmonary TB represents approximately 15% of cases, however this number has been gradually increasing in recent years [3]. Apart from the lungs, other organs are often affected. These concomitant pulmonary or extrapulmonary TB conditions are more difficult to cure [4, 5].

In some cases, there is simultaneity between lung and extrapulmonary forms, which is more frequent in individuals with acquired immunodeficiency syndrome (AIDS), with severe immune compromise limited to the context of deprivation and precarious access to health services [4, 5].

Analyzing concomitant TB considering the social context and how the space and its determinants act on the incidence of the disease are necessary aspects that can contribute to the qualification of the health services and to the advance of the equity in territories classified as critical. Several resources in the area of geography and exact sciences have made it possible to measure the degree of risk in a territory, to estimate temporal trends and make predictions. These approaches help health care workers and health services develop programs to avoid disease, measure progress, impact, and efficacy of existing preventative efforts [6].

It is accepted that TB incidence changes over time, thus these temporal trends are different, with respect to different geographical regions. Thus, these factors may provide additional information to help disease prevention, implement control measures, and tackle new health hazards [7].

To monitor emerging spatial patterns and temporal trends in disease risk, several spatiotemporal models for disease mapping have been proposed, including Spatial Variations in Temporal Trends (SVTT). SVTT identifies areas with specific epidemiological patterns/distributions, not explained by randomness. Equally, more sensitive technologies such as geo-statistical analyses are also helpful in supporting public health decisions, and implementing strategic interventions [7].

Despite the relevance of these different technologies for public health and surveillance, such approaches are under-used. Of the studies investigating territory disparity for concomitant TB burden, none used spatial variations to investigate temporal disease trends.

Thus, we analyzed variations in temporal trends, in areas at risk for concomitant TB. We sought to characterize clinical and epidemiological profiles of TB cases in a hyperendemic municipality in the Amazon region of Brazil.

## Methods

### Study setting

**Fig. 1:**
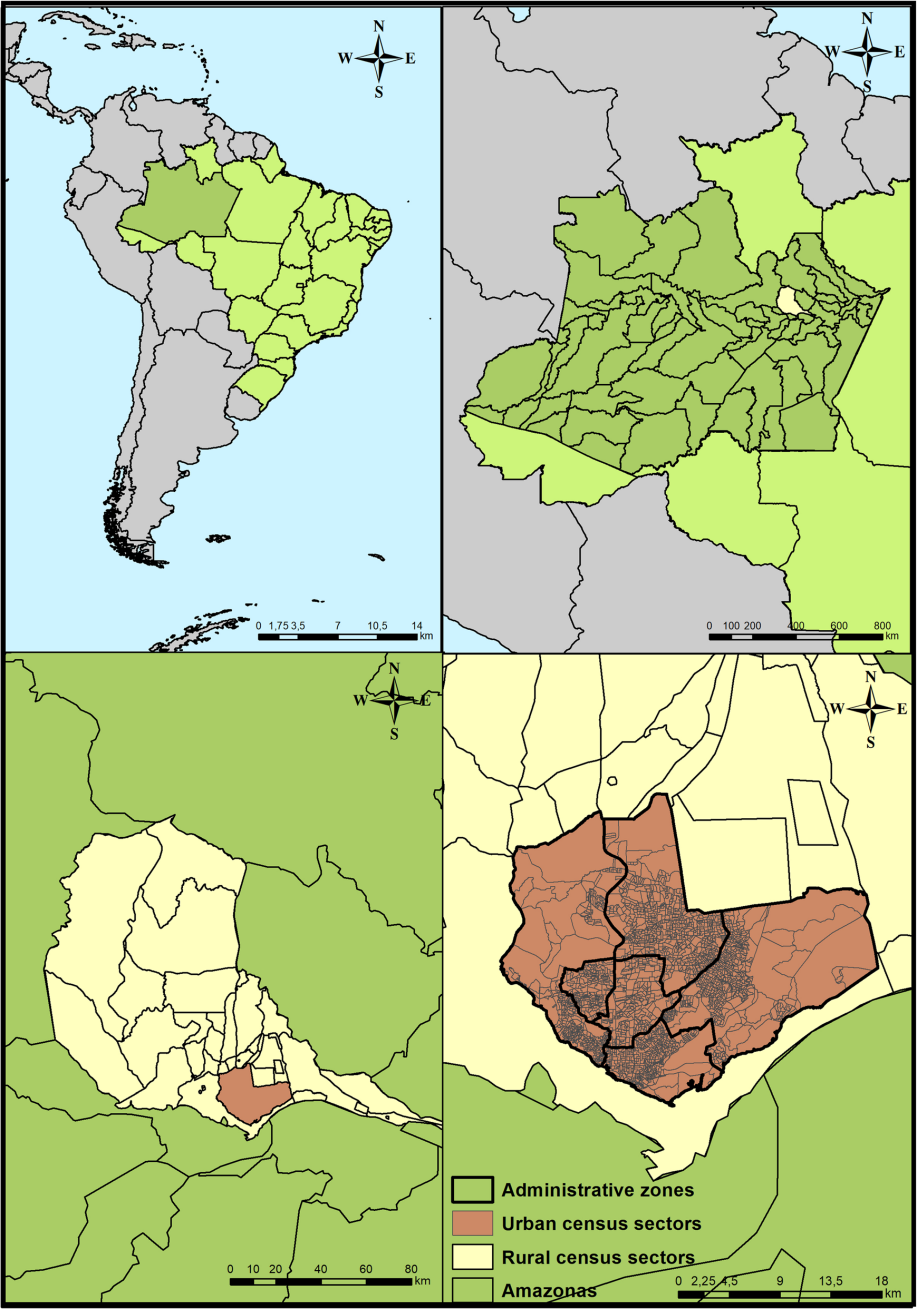
Geographical location of the Manaus municipality, Amazonas, Brazil

This was an ecological study [8] carried out in the Manaus municipality, capital of Amazonas, Amazon region, in northern Brazil. The area is located at 60^o^01’30“W longitude and 03^o^06’07”S latitude (Fig. 1), with an estimated population of 2 057 711 [9].

In terms of Brazilian epidemiology, Amazonas is unique because of high TB incidences in recent years. The state capital, Manaus leads TB incidence indices among state capitals, and is considered a hyperendemic TB municipality [10]. According to the Notifiable Diseases Information System (SINAN), in 2018, the municipality recorded an incidence coefficient of 102.6 TB cases/100 000 inhabitants. This was more than double Brazils coefficient i.e. 34.8 TB cases/100 000 inhabitants [10].

The municipality is geographically divided into 63 districts, distributed across six administrative zones: north, south, east, west, center west and center south. In addition, Manaus is segmented into 2461 census sectors, of which 2420 are urban and form part of this study [9].

### Population, information sources and selection criteria

Our study population consisted of concomitant pulmonary and extrapulmonary TB cases, registered at SINAN from January 1, 2009 to December 31, 2018. Data collection occurred in 2019 and was performed via the Management of Epidemiological Surveillance of the Municipal Health Secretariat of Manaus (SEMSA). We excluded duplicate cases; the most current record was considered.

For the georeferencing stage of spatial analysis, only residents of urban areas were considered. Records with incomplete addresses were excluded as geographical coordinates could not be ascertained. Cases reported in prisons or residents outside the municipality were excluded.

### Data analysis

First, cases of concomitant TB were grouped by month of notification and the monthly incidence rate was calculated. This procedure was performed using Microsoft Office Professional Plus 2016, in Excel (Microsoft Corp., Redmond, WA, USA).

To estimate the time trend over the study period of TB cases concomitant with cases and calculated monthly rates, we used the decomposition method called Seasonal-Trend by Loess (STL), which is based on a locally weighted regression [11]. Graphs for these cases, monthly incidence rates and time trend estimations were all performed using RStudio software (RStudio Inc., Boston, MA, USA).

For spatial analysis stage, initially in the geo-referencing of cases of concomitant TB, Google Earth Pro® software (Google Inc., Mountain View, CA, USA) was used to obtain geographical coordinates for residential addresses (latitude and longitude). The Manaus digital grid was obtained from the Brazilian Institute of Geography and Statistics (IBGE) open access website (https://www.ibge.gov.br/).

For risk cluster detection, the purely spatial scanning statistical technique developed by Kulldorff and Nagarwalla (1995) [12], also known as Scan statistics, was performed using SatScan software, version 9.6 (developed by Martin Kulldorff together with Information Management Services Inc. New York City, NY, USA). Risk clusters were identified through circular windows, with a variable radius around the centroid of each census sector in the municipality under analysis. For this study, each window tested our formal hypothesis, i.e. H0: there were no clusters in regions or areas of the analyzed municipality, and H1: in a certain area, the probability of occurring cases is lower or higher than in other regions of the municipality.

For the identification of spatial clusters, the following criteria were used in the analysis software: Poisson’s discrete model, the clusters cannot be geographically overlapped, maximum size of the clusters equal to 50% of the exposed population, clusters with circular shape and 999 replications following the Monte Carlo criteria [13].

The relative risk (RR) of each identified cluster was calculated which is the probability of the event (concomitant TB) occurring in the exposed group (cluster) compared to the unexposed group (outside the cluster). For interpretation purposes, RR = 1 means that there is no statistically significant difference between the exposed and unexposed groups; if RR < 1, this tended towards zero, i.e. low risk (or protection), whereas RR > 1 denoted a risk area. Clusters with a *P*-value < 0.05 were considered statistically significant, and the 95% confidence interval (*CI*) was estimated.

Scanning statistics also made it possible to incorporate temporal factors, in which the interest is in the identification of event clusters, in the case of this research, cases of concomitant TB, which have occurred in space and time, simultaneously. Thus, the same criteria mentioned above were used in the analysis software adding precision of time in day, month and year and time period between 2009 and 2018. RR was also calculated for the clusters identified in the space-time analysis.

The technique SVTT was used for the detection and inference of clusters, with significantly different time trends [7]. In this analysis, the scan window is of a purely spatial nature, however the time trend is calculated, both inside and outside the scan window, for each location and size [14].

When a difference in the temporal trend between the internal and external areas is detected, its statistical significance is calculated. Each window tested the following hypotheses, H0: the time trends are the same in all areas, and H1: the trends are different. Thus, the clusters detected in this analysis indicate areas whose temporal tendency is likely (Log likelihood ratio, LLR) to be different from what happens outside the cluster [7], adopting a type I error (*P* < 0.05) for statistically significant.

The results indicated an Internal Temporal Trend (ITT), which consisted of the degree of growth or decrease of the event within the cluster, and an External Temporal Trend (ETT), which corresponded to the trend of all other areas that do not belong to this cluster in question. Therefore, what is statistically significant in this analysis is the ITT and ETT found in that area [7].

As analysis criteria, it was also used Poisson discrete probability model, with time incorporated as an independent variable, the number of events as dependent variables, and the time for changing the population size, as an offset. The analysis was conditioned to the general trend, since the main objective was to verify differences in trends between areas [15]. Thematic maps were created using ArcGis software version 10.6 (ESRI Inc., Redlands, CA, USA).

We also performed descriptive analyses (absolute and relative frequencies) of the notified cases within each cluster, identified by spatial scanning, temporal space and SVTT as well as for all cases of concomitant TB reported during the study period using IBM SPSS® 25.0 software (IBM Corp., Armonk, NY, USA). The variables selected for descriptive statistics included: socio-demographic profiles (age, gender, race/color, education, employment status and beneficiary of an income transfer program) and clinical and operational profiles (case type, registration type, disease form, clinical results, TB-HIV co-infection status, TB-diabetes status, alcoholism, drug addiction, smoking and performing Directly Observed Treatment [DOT]).

### Ethical considerations

The study was approved by the Research Ethics Committee of the Ribeirão Preto School of Nursing at the University of São Paulo. The Certificate of Presentation for Ethical Appreciation (CAAE) No. 09994619.7.0000.5393.

## Results

### Characteristics of data from the historical series

Between 2009 and 2018, approximately 21 650 TB cases were reported in Manaus, of which 873 (4.0%) were concomitant TB. The age range of those affected by both disease forms was 1–94 years old, with an average age of 36.6 years. The median age was 34 years (standard deviation [SD] ± 14.2 years).

**Fig. 2:**
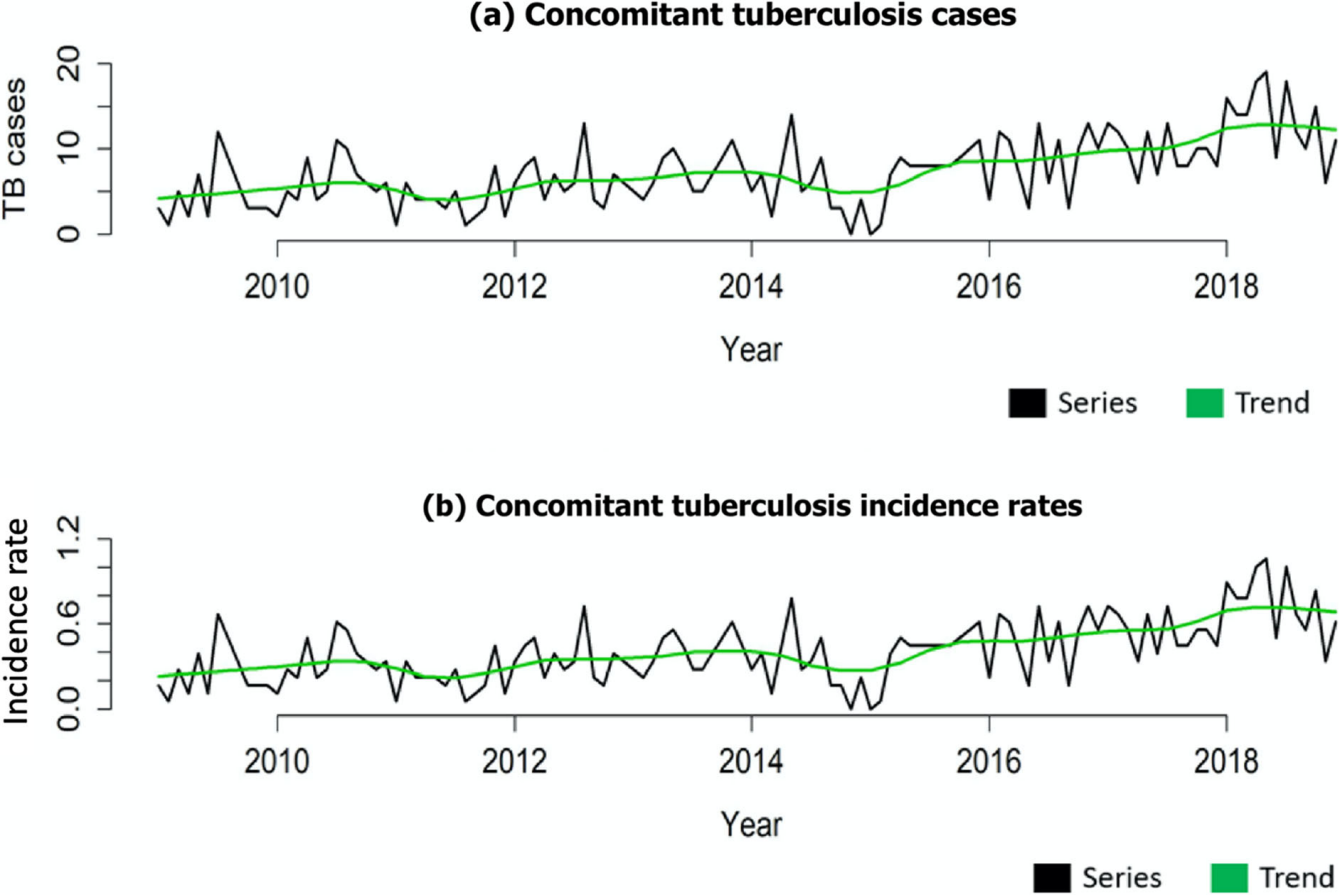
Time series and trends of cases and concomitant tuberculosis incidence rate in Manaus, Amazonas, Brazil (2009–2018)

From Fig. 2a, the evolution of TB cases was evident, and from Fig. 2b, we identified the evolution of concomitant TB incidence rates over the study period. In addition, a growing trend of cases and rates over the period was noted from these figures.

### Spatial risk and spatial-time risk to concomitant TB

In the spatial analysis of the 873 cases, 27 were excluded because they were prison diagnoses, and residents in rural areas and/or address fields not completed during notification. Thus, 846 cases proceeded to the next stage, where 685 (80.9%) were geo-referenced. The losses occurred due to the failure to fill in addresses and incorrect addresses.

**Fig. 3:**
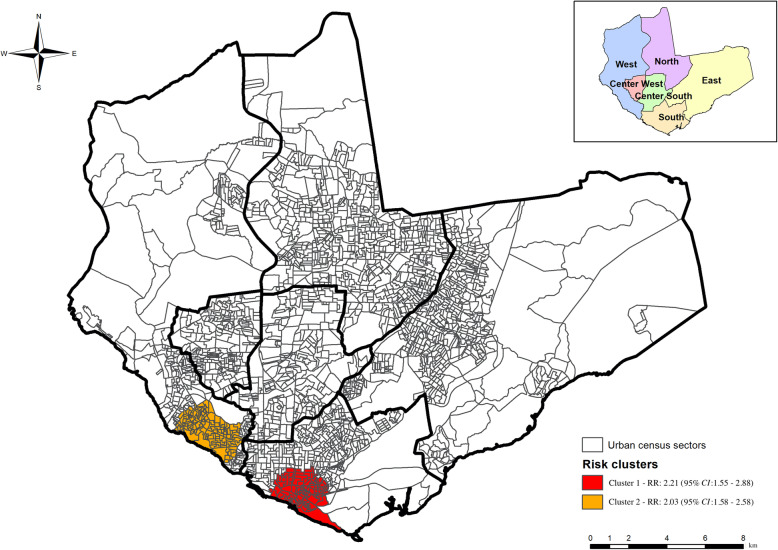
Spatial risk areas for concomitant pulmonary and extrapulmonary tuberculosis occurrences in Manaus, Amazonas, Brazil

Using purely spatial scanning statistics, two statistically significant (*P* < 0.05) spatial clusters were identified, agreeing with our alternative hypothesis (H1) that disease risk was higher inside these clusters than outside. Cluster 1 (*P* = 0.0031, RR = 2.21, 95% *CI*: 1.57–2.88) was composed of 110 census sectors in the south zone, comprising 73 390 inhabitants, 58 TB cases and an average rate of 7.9 cases/100 000 inhabitants. Cluster 2 (*P* = 0.0029, RR = 2.03, 95% *CI*: 1.58–2.58) was composed of 127 census sectors in the west zone, comprising 101 179 inhabitants, 77 TB cases and an average rate of 7.2 cases/100 000 inhabitants (Fig. 3).

**Fig. 4:**
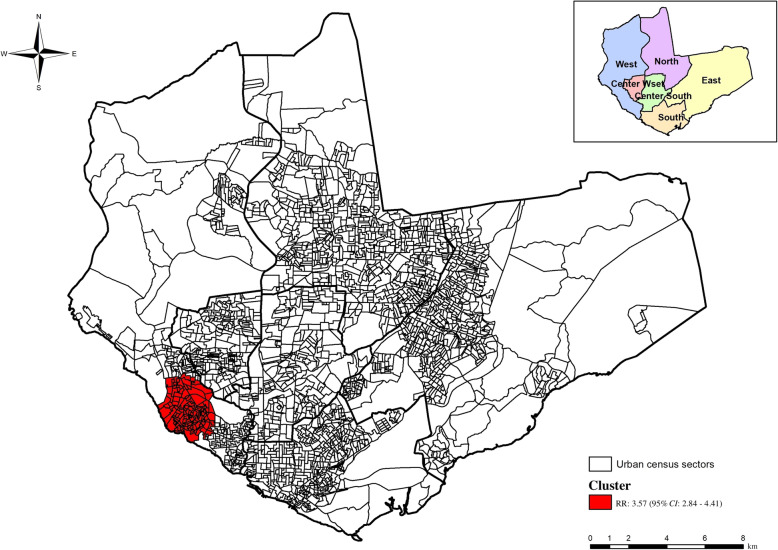
Spatial-time risk area for concomitant pulmonary and extrapulmonary tuberculosis occurrence in Manaus, Amazonas, Brazil

Using space-time scanning, we identified a risk cluster (*P* = 0.014, RR = 3.57, 95% *CI*: 2.84–4.41) between 2017 and 2018. The cluster was composed of 123 census sectors in the west zone, comprising 103 959 inhabitants and 58 confirmed TB cases (Fig. 4).

### Trends of concomitant TB

Using SVTT analysis from 2009 to 2018, the average population in census sectors of Manaus urban areas was 1 807 032. On average, four individuals were infected with concomitant pulmonary and extrapulmonary TB per 100 000 people per year, and the annual time trend increased by 9.7%.

**Fig. 5:**
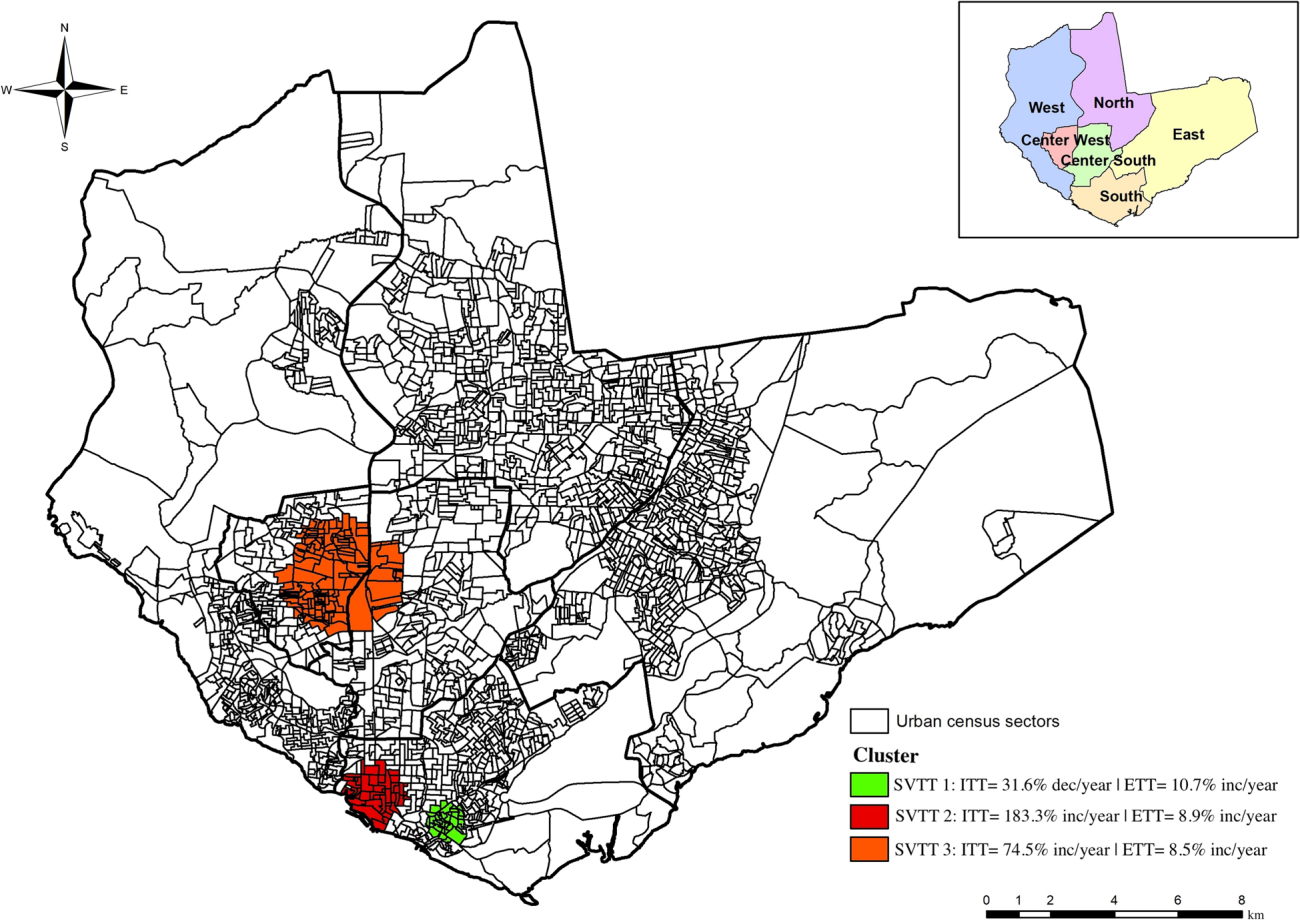
Clusters with a tendency to reduce or increase incidences of concomitant pulmonary and extrapulmonary tuberculosis in Manaus, Amazonas, Brazil. **ITT:** Internal Temporal Trend; **ETT:** External Temporal Trend; **dec:** decrease; **inc:** increase

Based on this data, three clusters with spatial variations were detected in the significant temporal trends (*P* < 0.05) (Fig. 5).

Cluster SVTT 1 (*P* = 0.042) was composed of 34 census sectors in the south zone, comprised 21 582 inhabitants, with 15 confirmed and eight expected TB cases. Cluster SVTT 2 (*P* = 0.046) was composed of 43 census sectors in the south zone, comprised 21 235 inhabitants, with 11 confirmed and eight TB expected cases. Cluster SVTT 3 (*P* = 0.036) was composed of 123 census sectors in the center west and central south zones, comprised 83 318 inhabitants, with 26 and 31.27 expected TB cases.

The ITT decreased 31.6% per year in Cluster SVTT 1, while in the clusters of SVTT 2 and SVTT 3, the ITT increased per year, respectively, 183.3% (highest ITT) and 74.5%; and the ETT increased per year 10.7, 8.9 and 8.5%, respectively for each cluster.

### Characteristics of cases in clusters

For each cluster detected by spatial, space-time and SVTT scanning techniques, we recorded case socio-demographic, clinical and operational characteristics (Table 1).

**Table 1:** Socio-demographic, clinical and operational characteristics of concomitant TB cases found in clusters in Manaus, Amazonas, Brazil (2009–2018)

Variables	Spatial Cluster 1 ***n*** = 58, ***n*** (%)	Spatial Cluster 2 ***n*** = 77, ***n*** (%)	Temporal Trend Cluster 1 ***n*** = 15, ***n*** (%)	Temporal Trend Cluster 2 ***n*** = 11, ***n*** (%)	Temporal Trend Cluster 3 ***n*** = 26, ***n*** (%)	Spatio-temporal Cluster 1 ***n*** = 58, ***n*** (%)	All cases ***n*** = 873, ***n*** (%)
**Socio-demographics**							
**Age**							
0–14 years	2 (3.4)	2 (2.6)	–	1 (9.1)	–	–	19 (1.9)
15–30 years	19 (32.8)	25 (32.5)	4 (26.7)	3 (27.3)	13 (50.0)	19 (32.8)	307 (35.3)
31–59 years	32 (55.2)	49 (63.6)	9 (60.0)	6 (54.5)	12 (46.2)	38 (65.5)	483 (55.3)
≥ 60 years	5 (8.6)	1 (1.3)	2 (13.3)	1 (9.1)	1 (3.8)	1 (1.7)	64 (7.4)
**Gender**							
Male	16 (72.4)	54 (70.1)	10 (66.7)	11 (100.0)	20 (76.9)	40 (69.0)	618 (70.8)
Female	42 (27.6)	23 (29.9)	5 (33.3)	–	6 (23.1)	18 (31.0)	255 (29.2)
**Race/color**							
Yellow	–	–	–	–	–	–	04 (0.5)
White	9 (15.5)	7 (9.1)	2 (13.3)	–	3 (11.5)	6 (10.3)	96 (11.0)
Brown	47 (81.0)	67 (87.0)	13 (86.7)	11 (100.0)	23 (88.5)	50 (86.2)	739 (84.7)
Black	–	2 (2.6)	–	–	–	2 (3.4)	19 (2.2)
Indigenous	1 (1.7)	1 (1.3)	–	–	–	–	05 (0.6)
Ignored	1 (1.7)	–	–	–	–	–	10 (1.1)
**Education**							
Illiterate	–	–	–	–	–	–	23 (2.6)
Primary	18 (31.1)	24 (31.2)	4 (26.6)	1 (9.1)	5 (19.2)	19 (32.8)	299 (34.2)
Incomplete high school	3 (5.2)	10 (13.0)	1 (6.7)	1 (9.1)	5 (19.2)	6 (10.3)	89 (10.2)
Complete high school	25 (43.1)	27 (35.1)	9 (60.0)	5 (45.5)	12 (46.2)	24 (41.4)	254 (29.1)
Incomplete undergraduate	3 (5.2)	4 (5.2)	–	–	1 (3.8)	1 (1.7)	37 (4.2)
Complete undergraduate	3 (5.2)	2 (2.6)	–	2 (18.2)	3 (11.5)	1 (1.7)	30 (3.4)
Ignored	6 (10.3)	10 (13.0)	1 (6.7)	2 (18.2)	–	–	141 (16.2)
**Profession/Employment**							
Unemployed	3 (3.9)	9 (11.7)	1 (6.5)	–	1 (3.9)	6 (10.4)	18 (2.0)
Formal Work	4 (5.4)	5 (6.5)	3 (20.1)	–	1 (3.9)	2 (3.4)	47 (5.4)
Informal Work	10 (17.0)	10 (13.0)	3 (20.1)	–	3 (11.4)	6 (10.3)	156 (17.9)
Ignored	41 (70.7)	53 (68.8)	8 (53.3)	11 (100.0)	21 (80.8)	44 (75.9)	652 (74.7)
**Beneficiary cash transfer programs**							
No	20 (34.5)	29 (37.7)	2 (13.3)	10 (9.1)	19 (73.1)	28 (48.3)	421 (48.2)
Yes	1 (1.7)	5 (6.5)	–	–	–	4 (6.9)	25 (2.9)
Ignored	37 (63.8)	43 (55.8)	13 (86.7)	1 (90.9)	7 (26.9)	26 (44.8)	427 (48.9)
**Clinical-operational**							
**Type of case**							
New	56 (96.6)	69 (89.6)	15 (100.0)	10 (90.9)	25 (96.2)	52 (89.7)	808 (92.6)
Relapse	1 (1.7)	4 (5.2)	–	1 (9.1)	–	5 (8.6)	30 (3.4)
Retreatment	–	3 (3.9)	–	–	–	1 (1.7)	15 (1.7)
Ignored\others	1 (1.7)	1 (1.3)	–	–	1 (3.8)	–	02 (0.2)
**Outcome**							
Abandonment	5 (8.6)	11 (14.3)	2 (13.3)	–	(7.7)	7 (12.1)	02 (0.2)
Cure	37 (63.8)	39 (50.6)	7 (46.7)	6 (54.5)	(57.7)	28 (48.3)	469 (53.7)
Bankruptcy/Resistance	2 (3.4)	4 (5.2)	–	–	(3.8)	6 (10.3)	50 (5.7)
Diagnostic Change	1 (1.7)	4 (5.2)	1 (6.7)	–	(3.8)	2 (3.4)	34 (3.9)
Death from other causes	6 (10.3)	9 (11.7)	2 (13.3)	2 (18.2)	(11.5)	8 (13.8)	44 (5.0)
Death with TB as a basic cause	4 (6.9)	6 (7.8)	3 (20.0)	–	(3.8)	2 (3.4)	100 (11.5)
State/country Transfer	2 (3.4)	–	–	1 (9.1)	–	–	127 (14.5)
Ignored	1 (1.7)	4 (5.2)	–	2 (18.2)	(11.5)	5 (8.6)	47 (5.4)
**TB-HIV co-infection**							
No	21 (36.2)	28 (36.4)	6 (40.0)	1 (9.1)	9 (34.6)	18 (31.0)	214 (24.5)
Yes	36 (62.1)	49 (63.6)	8 (53.3)	9 (81.8)	17 (65.5)	40 (69.0)	540 (61.9)
Test not performed	–	–	–	–	–	–	103 (11.7)
Ignored	1 (1.7)	–	1 (6.7)	1 (9.1)	–	–	16 (1.9)
**TB-diabetes comorbidity**							
No	53 (91.4)	74 (96.1)	14 (93.3)	9 (81.8)	23 (88.5)	56 (96.6)	819 (93.8)
Yes	4 (6.9)	3 (3.9)	1 (6.7)	1 (9.1)	3 (11.5)	2 (3.4)	37 (4.2)
Ignored	1 (1.7)	–	–	1 (9.1)	–	–	17 (1.9)
**Alcoholism**							
No	48 (82.8)	60 (77.9)	12 (80.0)	9 (81.8)	19 (73.1)	42 (72.4)	704 (80.6)
Yes	7 (12.1)	15 (19.5)	2 (13.3)	1 (9.1)	7 (26.9)	14 (24.1)	140 (16.0)
Ignored	3 (5.2)	2 (2.6)	1 (6.7)	1 (9.1)	–	2 (3.4)	29 (3.3)
**Drug addiction**							
No	18 (31.0)	22 (28.6)	3 (20.0)	3 (27.3)	9 (34.6)	18 (31.0)	401 (45.9)
Yes	2 (65.5)	7 (9.1)	1 (6.7)	1 (9.1)	1 (3.8)	3 (5.2)	65 (7.4)
Ignored	38 (3.4)	48 (62.3)	11 (73.3)	7 (63.6)	16 (61.5)	37 (63.8)	407 (46.6)
**Smoking**							
No	16 (25.6)	25 (62.3)	2 (13.3)	3 (27.3)	10 (38.5)	20 (34.5)	388 (44.4)
Yes	4 (6.9)	4 (5.2)	2 (13.3)	1 (9.1)	–	1 (1.7)	80 (9.2)
Ignored	38 (65.5)	48 (62.3)	11 (73.3)	(7 63.6)	16 (61.5)	37 (63.8)	405 (46.4)
**Directly observed treatment**							
No	21 (36.2)	20 (26.0)	8 (53.3)	1 (9.1)	7 (26.9)	15 (25.9)	285 (32.6)
Yes	8 (13.8)	10 (13.0)	3 (20.0)	–	1 (3.8)	5 (8.6)	104 (11.9)
Ignored	29 (50.0)	47 (61.0)	4 (26.7)	10 (90.9)	18 (69.2)	38 (65.5)	484 (55.4)
**Clinical form**							
Intestinal	3 (5.1)	2 (2.6)	1 (6.7)	1 (9.1)	2 (7.6)	4 (8.0)	29 (3.3)
Peritoneal	–	2 (2.6)	–	–	–	1 (1.7)	07 (0.8)
Disseminated	–	1 (1.3)	–	–	–	1 (1.7)	05 (0.6)
Ganglionar	–	–	–	–	–	–	04 (0.5)
Abdominal	–	1 (1.3)	–	–	–	1 (1.7)	03 (0.4)
Pericardial	1 (1.7)	–	–	–	–	–	03 (0.4)
Cerebral	–	–	–	–	–	–	02 (0.3)
Others	2 (3.5)	–	1 (6.7)	–	1 (3.8)	–	10 (1.1)
Missing	58 (89.7)	71 (92.2)	13 (86.7)	10 (90.9)	23 (88.5)	51 (87.9)	810 (92.8)

Table 1**.** Socio-demographic, clinical and operational characteristics of concomitant tuberculosis cases found in clusters in Manaus, Amazonas, Brazil (2009–2018).

### Discussion

We analyzed variations in temporal trends in areas at risk for concomitant TB, and we also characterized the clinical and epidemiological profile of TB cases in Manaus, Amazonas, Brazil.

By analyzing historical concomitant TB cases and rates between 2009 and 2018, we observed a trend in disease growth in the Manaus municipality, reflecting huge challenges in meeting World Health Organization (WHO) goals for the elimination of TB [1]. This disease goes beyond biological boundaries, making it vital to discuss its social determinants, especially in regions of great inequality, e.g. some Brazilian regions [16, 17].

The literature shows that disordered population growth, high population concentrations in urban areas, unemployment and migratory movements are associated with TB progression. These are accompanied by impaired health care systems and treatment failures (abandonment and multidrug resistant tuberculosis [MDR-TB]), which contribute to increased concomitant TB incidences [18, 19]. In poor areas with limited health services, the increased incidence and impact of TB on local populations may be even greater, if social access to health care is not resolved [16, 20].

From our spatial analyses and scanning statistics, we verified the formation of clusters in areas where we can consider them as at risk for the occurrence and transmissibility of TB. It is important to highlight Cluster 1, with the highest RR (RR = 2.21), where census sectors were located in the south of the city. In terms of socio-demographic characteristics, this area has the oldest neighborhoods, and is where the waterway port is located. It has a high circulation of people, it is also a commercial center and it concentrates industrial activities related to the Manaus free trade zone [21].

This region also has a high population density, i.e. 286 400 inhabitants, with irregular occupations, where houses with poor sanitation are built on river banks and streams. Here the poverty is strongly marked, which may represent explanatory elements for the TB risk cluster in the scenario as this type of indicator proves to be sensitive regarding the definition of spaces of greatest social vulnerability to TB [17]. Manaus is characterized by high population concentrations and economic resources in Amazonas, where approximately 54% of the state’s population and > 70% of the TB cases registered in previous years are concentrated [22].

Spatial Cluster 2 (RR = 2.03), with census tracts in the western part of the municipality is characterized by expanding neighborhoods, and middle class residential condominiums. The area is prevalent with illegal occupations and disorderly population growth [23], thereby constituting potential risk factors for disease occurrence [24].

A spatiotemporal risk cluster (RR = 3.57) also appeared in western regions of the municipality between 2017 and 2018, and may have been related to improvements in health surveillance services, such as awareness campaigns, active case-finding and consequently, were more prone to disease compared to the west and south regions, assuming that other regions may have had underreported cases [25].

In line with spatial scans, the formation of SVTT clusters 1 and 2 (Fig. 5) was also observed in census tracts in the south when the SVTT method was applied, pointing to indications of a tendency to increase cases in these areas found to be at risk for the disease.

The south region, as well as having high numbers of neighborhoods and a greater population density, has precarious housing, where intimate and prolonged contact is greater in home environments, potentially facilitating *Bacilli* transmission for TB and leprosy [26]. A study focusing on the spatial distribution of TB in Manaus showed that the highest rates of disease incidences were in that region (south), which could relate these indexes to sociodemographic conditions such as the number of people per bedroom, unemployment rate and low proportion of families linked to the sewage system [27].

Our SVTT analysis also identified the SVTT 3 Cluster as a risk area, with an increased tendency for concomitant TB incidence. These census sectors included areas between the mid-west and mid-south zones, containing rich and developed areas, with the highest average income per capita [10]. Studies outlining health inequalities have indicated that areas with population groups with better socioeconomic status, have more resources to spend on health when compared with the poorest populations [28, 29].

In addition, increased financial conditions and individual behaviors, such as easier access to health services, in addition to bio-psychosocial factors, can also influence health disparities in certain areas of the same geographical space [29], constituting the hypotheses for the trend of an increase in the number of case reports, because in addition to socioeconomic conditions and the general health status of the population, variations in the incidence of TB are also determined by the performance of the disease control actions in each location.

In view of this, it is worth mentioning that the characteristics of the disordered urban and demographic expansion in Manaus over the years, have influenced not only its socioeconomic and environmental conditions, but also on the spread of neglected diseases such as TB in its most serious forms, because its occurrence is probably related to lifestyle and also the exposure of people to precarious conditions [25, 30]. Therefore, richer more developed areas may show increasing tendencies for case numbers in this region, since TB is a dynamic disease, influenced by individual space and characteristics.

For case characterization in clusters and in the total of cases registered in the municipality, individuals of productive age (15–59 years old) had the highest notification rates, with a higher prevalence in males. This may be explained by an increased exposure to disease risk factors e.g., exposure to unhealthy activities, corroborating epidemiological TB profile data from other settings [31–33].

It is noteworthy that despite Amazonas having the largest indigenous population in the country [10], in Manaus, the population majority describe themselves as brown, potentially explaining the higher TB rates in this population, across all clusters. In addition, indigenous populations are concentrated in municipality rural areas, and the interior. Another factor that could explain the high TB rates in the brown population are health indicator inequalities between these and black populations; living conditions are more precarious in brown populations, including lower incomes and limited access to health services; and it is known that TB is a disease with strong social determination [34].

In our study population, the outcome ‘cure’ was below that recommended by the Brazilian Ministry of Health, which determined a percentage of 85% of new cases cured, similar results were found in other studies in Brazil [35–37].

The disease treatment modality may have had a direct impact on this data, considering that the evidence showed decreased treatment abandonment in individuals who adhere to directly observed treatment-short course [37], in the municipality the data show a low rate in this modality, as the clinical management of TB cases outside the pulmonary parenchyma is centralized [22], that is, the treatment occurs in the self-administered modality, where the TB patient appears once every 30 days in the reference center for the monthly consultation and the professional of health delivers the medicines until the next consultation, representing a greater risk of abandonment, negatively impacting the cure of the disease.

In terms of risk factors, TB-HIV co-infection represented the majority of cases across all clusters, in agreement with the literature suggesting that TB was the most common opportunistic infection in individuals with HIV [38]. It was noteworthy that the growing increase in cases of people living with HIV, diagnosed with AIDS, impacted the increase in the number of cases of pulmonary and extrapulmonary TB. In recent years, countries with HIV epidemics have experienced increased TB incidences and death [39].

It is important to highlight failures in SINAN notifications for TB cases in this study. This showed a high index of important incomplete fields, i.e. data were missing at initial notification. These limitations reflected a lack of information for descriptive TB stages and geo-referencing. Despite these limitations, our results are useful for public health services, as they focus on priority populations and areas that require health interventions.

By identifying a risk cluster in a locality, showing the areas with the highest spatial risk for the occurrence of the disease, this study reinforces that control actions should focus on these regions, as they are possibly more vulnerable to infection by *Mycobacterium tuberculosis*, in order to be effective and can reflect throughout the municipality and indirectly in other regions of the country.

These areas have the highest risk and/or increasing trends for TB cases, and reflect the devastating influence of TB in Manaus. These areas are socially vulnerable, as clusters were markedly larger in these areas, reflecting reduced economic and social structures.

## Conclusions

This study presented the risk areas for concomitant TB in Manaus, the Brazilian Amazon region, identifying areas with a tendency for the disease to increase. The data show that the TB-determining factors for TB risk areas overlap in deprived urban areas and communities in the municipality, pointing to indications that the precarious living conditions can increase the occurrence of the disease. Therefore, if there is no reduction in social inequalities in Brazil, it will be unlikely to reach the targets agreed and launched with the END TB Strategy within the deadlines of international agreements.

In view of this, our study contributes to the public health field by generating key TB data that facilitates correct and prompt decision making, and formulating and adjusting public policies on TB control actions made by health services, defining strategies for prioritizing risk areas and creating mechanisms for agreed objectives and goals in the country.

## Data Availability

All the data supporting the study findings are within the manuscript. Additional detailed information and raw data will be shared upon request addressed to the corresponding author.

## Abbreviations

**TB**: Tuberculosis
**HIV**: Human immunodeficiency virus
**AIDS**: Acquired immunodeficiency syndrome
**SVTT**: Spatial Variations in Temporal Trends
**AM**: Amazonas
**SINAN**: Notifiable Diseases Information System
**IBGE**: Brazilian Institute of Geography and Statistics
**SEMSA**: Municipal Health Secretariat of Manaus
**ITT**: Internal Temporal Trend
**ETT**: External Temporal Trend
**DOT**: Directly observed treatment
**RR**: Relative risk
**LLR**: Log likelihood ratio
**CAAE**: Certificate of Presentation for Ethical Appreciation
**MFTZ**: Manaus Free Trade Zone
**MDR-TB**: Multidrug resistant tuberculosis
**SDGs**: Sustainable Development Goals
